# Teaching of sleep-disordered breathing in Brazilian dental schools: a nationwide study

**DOI:** 10.1186/s12909-026-09497-w

**Published:** 2026-06-03

**Authors:** Cristiane Pontes de Barros Leal, Flávia Martão Flório

**Affiliations:** 1 Faculdade São Leopoldo Mandic, Campinas, SP Brazil; 2 Department of Collective Health, Faculdade São Leopoldo Mandic, Rua José Rocha Junqueira, 13, Campinas, SP Brazil

**Keywords:** Obstructive sleep apnea, Dentistry, Sleep apnea syndromes, Dental education, Curriculum

## Abstract

**Background:**

Sleep-Disordered Breathing (SDB) encompasses a spectrum of conditions ranging from primary snoring to obstructive sleep apnea (OSA), a condition linked to significant cardiovascular, metabolic, and neurocognitive risks. Dentists are in a strategic position to identify SDB early, yet international studies highlight insufficient curricular coverage. In Brazil, little is known about how undergraduate dental programs incorporate SDB-related education.

**Methods:**

A nationwide cross-sectional study was conducted among the 238 Brazilian dental schools that participated in the 2019 National Student Performance Exam (ENADE). Two self-administered digital questionnaires were distributed: one to course coordinators and another to orthodontics professors. Data were collected from March to October 2022. Descriptive analyses were performed to assess curricular inclusion, workload, faculty training, perceived importance, and barriers to SDB education.

**Results:**

A total of 120 coordinators (response rate of 51.7%) and 161 professors (response rate of 86.6%) participated. Among coordinators, 70% reported that SDB was not included in the curriculum, 66.7% reported a lack of trained faculty, and none reported continuing education programs. Only 2.5% of institutions had a dental sleep medicine clinic, and the mean workload devoted to SDB content in the undergraduate curriculum was 5.6 h. Among professors, 93.2% considered SDB instruction important, but only 25.5% included it in their teaching. Few faculty members reported routinely asking about snoring or other sleep-related symptoms during clinical activities. Most (76.4%) classified dentists’ training in OSA as deficient, and 92.5% agreed that curricula should cover OSA.

**Conclusion:**

Despite recognition of its relevance, SDB education is largely absent from Brazilian dental curricula. Findings suggest a need for curricular reform, faculty development, and expanded clinical training opportunities. Future studies should triangulate data sources and explore innovative teaching strategies to address these curricular gaps.

**Supplementary Information:**

The online version contains supplementary material available at 10.1186/s12909-026-09497-w.

## Introduction

Sleep-disordered breathing (SDB) is a spectrum of conditions characterized by abnormal respiration during sleep. It includes primary snoring, upper airway resistance syndrome (UARS), obstructive sleep apnea (OSA), central and mixed apneas, and sleep-related alveolar hypoventilation [1–3]. Among these disorders, OSA stands out due to its high prevalence and strong association with adverse health outcomes, including cardiovascular disease, metabolic alterations, neurocognitive impairment, and reduced quality of life [4–9].

Epidemiological evidence indicates that approximately one billion adults worldwide may suffer from OSA, with nearly 425 million cases classified as moderate to severe [10]. In Brazil, population-based studies have reported a prevalence of 32.8% among adults [11].

Within this context, medical and dental organizations emphasized the importance of early identification and interdisciplinary management of SDB [12–14]. Dentists, in particular, are well positioned to screen for clinical signs and risk factors, given their routine assessment of the craniofacial complex and frequent contact with patients [5, 12, 15]. A recent Brazilian study demonstrated a significant association between malocclusion and sleep-disordered breathing in children, further highlighting the strategic role of dentists in early detection [16]. Their role as frontline professionals provides a unique opportunity for timely referral and collaborative care [17–19].

Despite the clinical significance of SDB, studies from several countries consistently reveal curricular gaps in dentistry programs, often characterized by limited curricular time, insufficiently trained faculty, and a lack of practical training opportunities [20–23]. For example, universities in North America, Europe, and Asia generally dedicate only a few hours to theoretical instruction, with little integration into clinical practice [22, 24–26].

In Brazil, national curricular guidelines [27] encourage comprehensive training to address health promotion and disease prevention. Internationally, several studies have systematically examined how sleep-disordered breathing (SDB) has been incorporated into dental curricula across North America, Europe, Asia, and Oceania [20–32]. However, to date, no published investigations have assessed this topic in Brazilian dental education, representing an important gap that this study aims to address.

Given the growing recognition of SDB as a public health issue and the strategic role of dentists in its detection, it is important to assess the current state of SDB teaching in Brazilian dental schools. This study aimed to evaluate how sleep-disordered breathing and obstructive sleep apnea are addressed in the undergraduate dental curriculum across Brazilian dental programs that had graduating students in the 2019 National Student Performance Exam (ENADE) cycle.

## Materials and methods

This nationwide cross-sectional study was approved by the Research Ethics Committee of *Faculdade São Leopoldo Mandic* (CAAE: 53058721.9.0000.5374) and conducted in accordance with the Declaration of Helsinki and Brazilian regulations for research involving human participants. Written informed consent was obtained electronically from all participants prior to enrollment. Data were collected anonymously, and no information that could directly identify participants or their institutions was stored, thereby ensuring confidentiality. The manuscript was prepared with attention to key STROBE recommendations for cross-sectional studies to improve reporting transparency.

### Study population and sampling

According to the 2019 ENADE - the nationwide assessment administered by the Brazilian Ministry of Education - Dentistry belongs to Cycle 3, which includes health and biological sciences programs evaluated every three years. In that edition, Brazil had 468 accredited undergraduate dental programs, of which 238 had graduating students, a prerequisite for taking the exam.

The sampling frame was defined by the 2019 ENADE and therefore included only undergraduate dental programs with graduating students eligible for assessment in that cycle. Consequently, programs without graduating students in 2019, including newly established programs, were not part of the ENADE-based sampling frame by design.

From these 238 eligible programs, 232 had publicly available contact information and were contacted (97.5%). No exclusion criteria were applied beyond the unavailability of public contact information. For each institution, course coordinators, defined as the academic leaders responsible for overseeing undergraduate dental programs, were invited to participate, as they were considered the most appropriate sources of institutional-level information on curricular structure and educational policies. In multi-campus universities, each campus was treated as a distinct institutional unit.

Coordinators who agreed to participate provided the contact information of their orthodontics professors, enabling the research team to send the faculty questionnaire directly. Orthodontics professors were selected because they commonly manage patients with craniofacial anomalies and functional airway issues associated with increased risk of SDB. Their teaching roles, which typically include craniofacial growth and airway function, also position them as key informants regarding the integration of SDB/OSA topics into dental curricula.

**Fig. 1:**
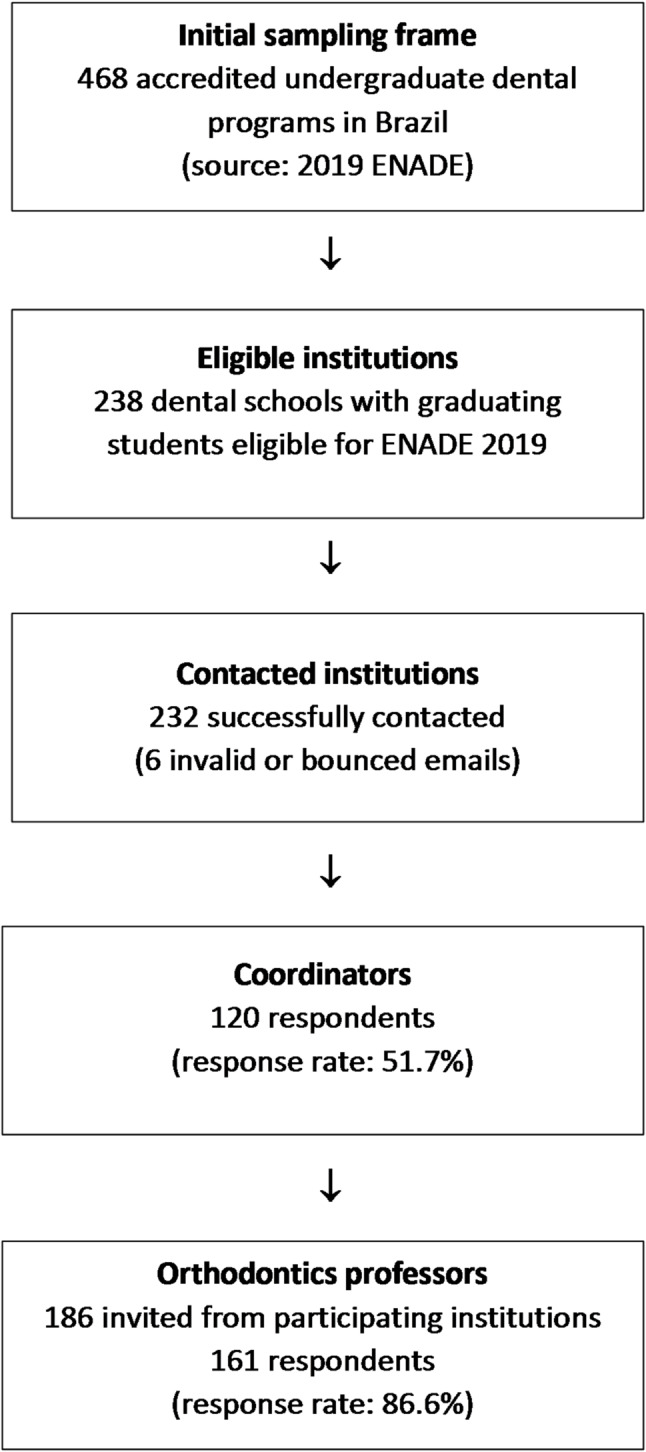
Flow diagram of participant inclusion and response rates. Each campus was considered a distinct institutional unit when multi-campus universities were identified. Percentages were calculated based on the number of institutions successfully contacted in each phase

In total, 120 course coordinators completed the survey, yielding a response rate of 51.7% (120/232). Based on the contact information supplied by these coordinators, 186 orthodontics professors were invited directly by email, and 161 responded, resulting in a response rate of 86.6% (161/186). These stages are summarized in Fig. 1.

### Data collection

Two self-administered digital questionnaires were developed using Google Forms, based on previously validated surveys in dental sleep medicine [22, 23, 29–36] and adapted to the context of Brazilian dental programs. Data were collected between March and October 2022.

The coordinator questionnaire (17 items, 11 analyzed in this study) included: (i) institutional characteristics (region, type, workload, course duration); (ii) curricular aspects (existence of a dental sleep medicine discipline, number of teaching hours, topics covered, availability of continuing education programs); (iii) faculty preparedness (availability of trained professors); and (iv) perceived barriers to implementation.

The professor questionnaire (56 items, 19 analyzed) was organized into blocks covering: (i) professional profile (years since graduation, years of teaching, orthodontics training, previous exposure to dental sleep medicine); (ii) perceptions of SDB teaching (importance, adequacy of training for general dentists and orthodontists); (iii) specific items on OSA (role of dentists in screening and referral, inquiry about snoring/bruxism, collaboration with sleep physicians, curricular inclusion); and (iv) willingness to participate in continuing education programs. Analyses were restricted to the items directly aligned with the study objectives and reported in Tables 1, 2, 3, 4 and 5.

**Table 1:** Characteristics of participating dental schools (coordinators’ survey, *n* = 120)

Question	Answer	Absolute frequency	Relative frequency
Institution’s geographic region	Northeastern	44	36.7%
	Southeastern	32	26.7%
	Southern	26	21.7%
	Northern	11	9.2%
	Midwestern	7	5.8%
Nature of the educational institution	Private	92	76.7%
	Public	28	23.3%
Workload of the dentistry course (hours)	Less than 4,000	2	1.7%
	4,000	30	25.0%
	Between 4,001 and 5,000	78	65.0%
	More than 5,000	8	6.7%
	Did not answer	2	1.7%
Existence (years) of the dentistry course	1 to 5	51	42.5%
	6 to 10	17	14.2%
	11 to 15	11	9.2%
	16 to 20	8	6.7%
	More than 20	33	27.5%

**Table 2:** Inclusion of SDB topics in undergraduate curricula: coordinators’ responses (*n* = 120)

Question	Answer	Absolute frequency	Relative frequency
Is there a dental sleep medicine clinic at the Institution?	No	117	97.5%
	Yes	3	2.5%
How many hours of theoretical or clinical classes are devoted to teaching sleep disorders / dental sleep medicine topics in the dentistry course?	0	31	25.8%
	1 to 8	49	40.8%
	> 8	8	6.7%
	> 24	1	0.8%
	Did not answer	31	25.8%
Does any department/discipline of the institution include the study of sleep disorders/dental sleep medicine?	No	84	70.0%
	Yes	36	30.0%
Does the institution have a faculty trained in sleep disorders/dental sleep medicine?	No	80	66.7%
	Yes	39	32.5%
	Did not answer	1	0.8%
Does the institution have continuing education programs on sleep disorders/dental sleep medicine at the undergraduate level?	No	118	98.0%
	Yes	0	0.0%
	Did not answer	2	1.7%
What challenges were faced to implement the study on sleep disorders/dental sleep medicine at the institution?	Lack of trained faculty in dental sleep medicine	37	30.8%
	Lack of administrative approval / physical space / institutional support	23	19.0%
	No challenges	28	23.3%
	Did not answer	38	31.7%
What topics related to dental sleep medicine are covered?	Sleep bruxism	71	59.2%
	Obstructive sleep apnea	55	45.9%
	Primary snoring	23	19.2%
	REM behavior disorder	22	18.4%
	Insomnia	19	15.8%
	Restless leg syndrome	8	6.7%
	Did not answer	46	38.3%

Percentages may exceed 100% because multiple responses were permitted

*SDB* Sleep-disordered breathing

**Table 3:** General characteristics of orthodontics professors (*n* = 161)

Question	Mean (standard deviation)	Median (minimum; maximum)
Time since graduation from the dentistry college (years)	23.4 (9.1)	24.0 (4.0; 44.0)
Time as an orthodontics specialist (years)	17.0 (8.4)	16.0 (1.0; 40.0)
Time as a professor of orthodontics, or of another discipline that includes orthodontics (years)	12.2 (8.5)	10.0 (1.5; 40.0)
Workload (hours) devoted to SDB/dental sleep medicine content in the undergraduate course	5.6 (9.1)	4.0 (0.0; 60.0)

Values represent hours reportedly devoted to SDB/dental sleep medicine content, which may or may not correspond to a standalone discipline

**Table 4:** Professors’ responses regarding dental sleep medicine teaching (*n* = 161)

Question	Answer	Absolute frequency	Relative frequency
Training in dental sleep medicine.	Lectures	70	43.5%
	Extension course with at least 30 h	24	14.9%
	Training	11	6.8%
	Other forms of learning	8	5.0%
	None	18	11.2%
	Did not answer	30	18.6%
Are there approaches to sleep disorders in the subject taught in the undergraduate course?	No	116	72.0%
	Yes	41	25.5%
	Did not answer	4	2.5%
How would you classify the importance of teaching sleep disorders in the undergraduate course?	Not important	3	1.9%
	Important	74	46.0%
	Very important	76	47.2%
	Did not answer	8	4.9%
How would you evaluate the training of dentists in understanding OSA?	Deficient	123	76.4%
	Regular	30	18.6%
	Very good	6	3.7%
	Did not answer	2	1.2%
How would you evaluate the training of orthodontists to understand OSA?	Deficient	83	51.6%
	Regular	67	41.6%
	Very good	8	5.0%
	Did not answer	3	1.9%
Have you ever referred an adult to a sleep doctor for suspected OSA?	No	39	24.2%
	Yes	118	73.3%
	Did not answer	4	2.5%
Have you ever referred a child to a sleep doctor for suspected OSA?	No	41	25.5%
	Yes	117	72.7%
	Did not answer	3	1.9%
Would you be interested in attending a course on dental management of sleep-related oral diseases?	No	22	13.7%
	Yes	138	85.7%
	Did not answer	1	0.6%

Response options are displayed exactly as presented in the questionnaire

*OSA* Obstructive sleep apnea

**Table 5:** Professors’ attitudes and perceptions toward SDB teaching (*n* = 161)

	Strongly agree	Agree	No opinion on the subject	Disagree	Strongly disagree	Did not answer
	*n* (%)	*n* (%)	*n* (%)	*n* (%)	*n* (%)	*n* (%)
Dentists play an important role in diagnosing and treating OSA	56 (34.8)	94 (58.4)	10 (6.2)	0 (0.0)	0 (0.0)	1 (0.6)
When dentists identify bruxism, they should inquire the patient about snoring and OSA	37 (23.0)	85 (52.8)	32 (19.9)	5 (3.1)	0 (0.0)	2 (1.2)
The dental curriculum in undergraduate courses should include information about OSA and the role of the dentist on this health problem	54 (33.5)	95 (59.0)	9 (5.6)	2 (1.2)	0 (0.0)	1 (0.6)
Screening the patient for OSA should be required in the dental clinical examination	41 (25.5)	103 (64.0)	11 (6.8)	4 (2.5)	0 (0.0)	2 (1.2)
Dentists and sleep doctors must deal with OSA patients together	71 (44.1)	84 (52.2)	4 (2.5)	0 (0.0)	0 (0.0)	2 (1.2)
If dentists find abnormal oral anatomical structures, they should perform further investigation for OSA and refer the patient to a sleep doctor	51 (31.7)	90 (55.9)	13 (8.1)	5 (3.1)	1 (0.6)	1 (0.6)

*SDB* Sleep-disordered breathing, *OSA* Obstructive sleep apnea

For this study, ‘theoretical inclusion’ referred to at least one lecture or theoretical module addressing SDB/OSA. ‘Practical activities’ included case discussions, patient evaluations, or clinical-laboratory exercises related to sleep-disordered breathing.

The instruments were further refined for this study as descriptive tools and reviewed by the research team to ensure conceptual clarity, cultural adequacy, and contextual relevance. They were pilot-tested with a small group of dental faculty (*n* = 5) to refine wording and comprehensibility, and revisions were made based on the feedback received. The pilot participants were not included in the final sample. No formal psychometric validation (e.g., internal consistency or reliability indices) was performed. The complete questionnaires, including all items and response options, are available in Supplementary Files S1 (Coordinator Questionnaire) and S2 (Professor Questionnaire), together with their English translations.

### Data analysis

Data were exported to Microsoft Excel and analyzed using descriptive statistics. Categorical variables were summarized as absolute and relative frequencies, whereas quantitative variables were expressed as means, medians, standard deviations, and ranges. Given the exploratory nature of this nationwide survey and the categorical structure of most variables, analyses were limited to descriptive statistics to map the current landscape of SDB teaching.

For items allowing more than one response (e.g., “Challenges”), results were calculated as the percentage of respondents, with multiple responses permitted; this is indicated in the corresponding table footnotes. For item-level missingness, valid response categories (e.g., “0 hours”) were analyzed separately from item nonresponse (e.g., “Did not answer”), which was coded as missing data and reported separately.

Data cleaning consisted of organizing and standardizing response categories for tabulation, including separate coding of item nonresponse (e.g., “Did not answer”). The analyses focused on overall descriptive estimates; subgroup comparisons (e.g., by institution type or geographic region) were not performed, as the primary aim was to describe the overall curricular landscape rather than testing between-group differences.

## Results

A total of 120 course coordinators participated, most of them from private institutions (76.7%) and located in the Northeast region (36.7%). Only 2.5% of dental schools reported having a dental sleep medicine clinic, and 70% indicated that no department or discipline was dedicated to SDB. Furthermore, 66.7% reported a lack of trained faculty. When present, the curricular workload devoted to SDB ranged from 1 to 24 h and was predominantly theoretical (Table 1). The most frequently covered topics were sleep bruxism (59.2%) and OSA (45.9%) (Table 2). Item-level nonresponse was observed across multiple items; therefore, “0 hours” was treated as a valid response category, whereas “Did not answer” was coded as missing data and reported separately.

Among the 161 orthodontics professors who responded, the mean time since graduation was 23.4 years, and the average workload devoted to SDB topics was only 5.6 h (Table 3). Most professors (93.2%) considered SDB teaching important or very important, yet only 25.5% reported addressing the subject in their courses. Training in dental sleep medicine was limited: 43.5% had only attended lectures, and 11.2% reported no prior training (Table 4).

There was a notable gap between perception and practice. Although 76.4% of professors classified general dentists’ training in OSA as deficient, and 92.5% agreed that curricula should include OSA content, few reported effective integration of this subject into their teaching. Results from Table 5 reinforce this paradox: the vast majority strongly agreed or agreed that dentists should play an important role in diagnosing and treating OSA (93.2%), that clinical screening for OSA should be part of routine dental examination (89.5%), and that dentists should collaborate with physicians in managing affected patients (96.3%). However, despite this consensus, curricular implementation remains limited, and only a minority of professors consistently inquire about snoring or sleep-related symptoms in their clinical practice.

Overall, both coordinators and professors recognized the importance of SDB in dentistry but reported insufficient curricular inclusion, lack of qualified faculty, and limited workload. These findings highlight a disconnect between perceived relevance and actual implementation, reinforcing the existence of substantial curricular gaps in Brazilian dental schools.

## Discussion

### Insufficient SDB teaching integration and implementation challenges

The inclusion of SDB teaching in Brazilian dental schools was found to be inadequate. Given the cross-sectional descriptive design, the findings should be interpreted as an overview of current teaching practices rather than evidence of causal relationships. Most institutions reported the absence of a department or discipline specifically dedicated to this subject, and professors often lacked specialized training to educate undergraduate students. Furthermore, only 2.5% of colleges reported having a dental sleep medicine clinic, indicating the limited availability of practical resources for students. Among professors, only 25% acknowledged addressing the topic, devoting on average 5.6 h of the course to its instruction. These findings suggest that SDB teaching remains limited in scope and insufficiently integrated with clinical practice. Therefore, the role of dentists in identifying and referring patients with SDB may not be adequately addressed within the current curricular content.

SDB teaching also faces additional challenges, such as lack of trained faculty and insufficient institutional support. According to course coordinators, 30.8% believed that the main reason for not implementing a specific discipline on SDB in undergraduate courses was the shortage of qualified professionals. These findings may suggest an association between the absence of sleep clinics and the lack of trained professors. Evidence from the US and Canada also shows that universities without sleep clinics usually lack qualified faculty, and even in those with such clinics, integrating dental sleep medicine into other disciplines remains difficult [23]. This pattern is consistent with the possibility that limited institutional infrastructure, such as the absence of a dental sleep medicine clinic, may be associated with the shortage of trained professors observed in the present study; however, causal inferences cannot be made given the descriptive design.

### Role of general dentists in SDB management

Despite the challenges, the role of general dentists in managing SDB is fundamental and well documented. The clinical practice guidelines of the American Academy of Dental Sleep Medicine [12] clearly highlight the importance of general dentists in screening and referring patients with SDB. In this role, dentists collaborate with physicians in the early identification of OSA, using diagnostic tools that range from simple questions about snoring, joint pain, and bruxism to the application of specific questionnaires assessing the airway, craniofacial features, and occlusion [12]. In our study, most professors agreed that dentists and physicians should work together in the management of patients with OSA and recognized the importance of dentists in diagnosis and treatment. This collaborative perspective is further supported by the literature that associates teaching on bruxism and snoring with the broader field of SDB [4, 5]. The coordinators’ responses also indicate that the main topics currently addressed in dental sleep medicine are sleep bruxism (59.2%), OSA (45.9%), and primary snoring (19.2%). However, the insufficient workload devoted to general dentists, coupled with the lack of correlation with clinical practice, may reduce opportunities for students to recognize the importance of including these aspects during patient anamnesis.

The strategic position of dentists in SDB identification is further supported by recent Brazilian research demonstrating significant associations between malocclusion and sleep-disordered breathing in pediatric populations [16]. Such findings support the importance of incorporating SDB education across multiple dental specialties, particularly orthodontics and pediatric dentistry, as these professionals are ideally positioned to identify early signs during routine clinical examinations.

### Current educational practices and perceptions

To further underscore the need for adequate SDB education, it is important to consider the current educational practices in Brazil and the perceptions of professors. Most professors reported referring both adults and children with suspected OSA to specialized physicians. However, routine screening behaviors were less consistently reported in clinical teaching. Only 58.4% of professors reported investigating OSA in patients with a history of snoring, and 77.6% indicated that they asked about sleep history after observing tooth wear. This gap suggests that referral practices may not be consistently accompanied by systematic screening approaches during routine dental care.

With respect to the early correlation between snoring and apnea [1, 3], a study conducted in Italy and Spain with children confirmed the high prevalence of snoring and showed that the risk of developing SDB in the presence of snoring was four times higher, making it the most prevalent symptom [5]. Taken together, these findings suggest that snoring and other sleep-related symptoms may warrant more consistent investigation as part of routine dental history-taking.

### Growing interest and importance of OSA education

As the dental community increasingly recognizes the importance of managing OSA, interest in integrating this topic into the dental curriculum has grown. Most professors (87.6%) agreed that when dentists identify abnormal oral anatomical structures, they should perform a more in-depth investigation of OSA and refer the patient to a sleep specialist. Features such as atretic palate, crossbite, severe crowding, micrognathia, and retrognathia are already part of standard dental diagnoses and position dentists as potential sentinels for the early detection of OSA [8, 13, 14].

In recent years, dentists have demonstrated growing concern about OSA, and the dental community has increasingly acknowledged it as a serious health problem requiring early identification [17, 37, 38]. In the present study, 93.2% of professors classified SDB teaching at the undergraduate level as important or very important, and 92.5% agreed that dental curricula should include OSA-related content. Nevertheless, despite recognizing its relevance, most professors do not address it in their courses.

One of the main barriers lies in time constraints: professors face difficulties introducing new content due to preset curricula and restricted workloads. Thus, although they acknowledge the importance of OSA, a gap remains between awareness and effective teaching. Furthermore, early diagnosis of OSA requires practical training opportunities within educational institutions. According to the national curricular guidelines for dentistry, professionals must act based on clinical and epidemiological recognition of health vulnerabilities. Therefore, the training of generalist dentists may benefit from a specific workload dedicated to OSA [27]. Additionally, continuing education courses should be available; however, none of the institutions evaluated in this study currently offered such opportunities.

### Curricular gaps and recommendations

Curricular gaps in SDB teaching remain evident. A study evaluating the curricula of 409 dental schools across 12 countries reported that 27% of programs did not address SDB [35]. Unlike Brazil, where dentistry is a standalone undergraduate program, in many of these countries dentistry is offered as a postgraduate course integrated within the medical curriculum [36]. In the Middle East, a survey of undergraduate students found that only 23% of colleges included the topic, with an average of just 1.2 h dedicated to it [25]. Similarly, in Australia and New Zealand, course coordinators reported an average of 4.5 h dedicated to SDB throughout the curriculum [31]. In Japan, a study covering 29 dental schools (89.7% of the total) found an average of only 3.8 h, predominantly theoretical; moreover, only 11.5% of professors reported clinical-laboratory experience [32].

In the present study, 25.8% of coordinators reported no theoretical or clinical workload devoted to SDB or dental sleep medicine, while another 25.8% did not answer this question. Such findings may reflect a lack of information or awareness about the inclusion of this subject within their curricula, thereby potentially increasing the frequency of these gaps. Among those who did report offering SDB content, the average workload was 5.6 h, predominantly theoretical. These results suggest that the limited or absent theoretical workload observed in Brazil, as well as in other countries, is insufficient to ensure proper clinical practice.

### Professors’ opinions on screening and training

In a context of insufficient training, a notable finding was that 89.5% of professors stated that screening for OSA should be a mandatory component of clinical dental examinations. The interest in managing these patients was also evident, as 85.7% of professors expressed a desire to attend courses on the dental management of sleep-related oral diseases. Similar results were reported in India, where despite poor training, 90% of general dentists expressed interest in learning more about OSA and acknowledged their responsibility in diagnosing and treating it [30]. In Finland, 83% of dentists interviewed agreed that they should be aware of SDB diagnosis, but only 34% reported having received any training on the subject during their undergraduate studies [17]. These findings reinforce the importance of aligning professionals’ willingness to learn more about OSA with the integration of this topic into the dental curriculum.

A meaningful next step would be to align the recognized importance of OSA with the implementation of earlier and more structured approaches to SDB diagnosis within dental training and practice. These findings highlight the importance of establishing earlier screening and referral pathways in dental settings, particularly in pediatric care, to prevent undiagnosed cases from persisting into adulthood. Unfortunately, in current practice, SDB symptoms may persist for years before the first diagnosis is made, even though the condition is strongly associated with multiple comorbidities that negatively impact both health and interpersonal relationships [15].

### Training and interdisciplinary treatment

Undergraduate training should aim to better equip students to identify respiratory disorders in patients. In Canada, the lack of clear guidance on the interdisciplinary aspect of treatment led the Canadian Sleep Society and the Canadian Academy of Dental Sleep Medicine to develop a document defining the limits and responsibilities of dental surgeons in managing OSA. These include recognizing SDB symptoms, referring patients to specialists when the disorder is suspected, and understanding comorbidities related to SDB, such as bruxism and orofacial pain [39].

In Saudi Arabia, a recent survey with dental surgeons assessing knowledge and attitudes toward OSA found that 63.35% reported training deficits, which contributed to a negative attitude toward the relevance of screening OSA patients. Conversely, dentists with greater knowledge were more likely to value the importance of this diagnosis [19]. In Brazil, the predominance of theoretical over practical approaches may limit professionals’ preparedness to understand the scope and severity of OSA. This may partly account for why, in the present study, 76.4% of professors rated the training of Brazilian dentists as deficient.

In the United States, studies have shown a growing interest among universities and general dentists in OSA, but they also emphasize that dentists are not being adequately prepared to manage respiratory disorders, mainly due to gaps in the curriculum [20, 23, 26, 29, 33]. The literature further highlights the importance of having qualified teaching staff, adequate laboratories, and comprehensive theoretical and practical courses; otherwise, the training of future dentists remains incomplete [26]. In our study, respondents reported not only a shortage of qualified professors, but also the lack of institutional approval or administrative support (10%) and insufficient physical space (5%) as barriers to implementing SDB teaching. The limited adherence of coordinators to this issue may indicate that curricular restructuring could be considered to strengthen SDB teaching. A promising example comes from the Netherlands, where some universities have begun a structural movement to include the diagnosis and treatment of SDB in dental schools, through the introduction of relevant disciplines and the establishment of specific clinics and facilities dedicated to appropriate examinations [3].

### Orthodontics and SDB

Orthodontics professors were included in the second stage of the study because this specialty treats patients at high risk of developing SDB [9, 13, 38, 40, 41]. Previous studies have shown that 7%–11% of orthodontic patients present a significant risk for some form of sleep disorder [7, 41], and orthodontics routinely diagnoses and treats problems that may ultimately lead to OSA, such as abnormal occlusal and craniofacial features [9, 13, 38, 40, 42]. In addition to clinical evaluations of the face and occlusion, commonly performed in orthodontic practice, diagnostic exams such as cephalometric radiography and cone-beam computed tomography (CBCT) are also useful tools for analyzing the upper airway and detecting potential reductions in its volume [13, 37, 43–46].

Therefore, orthodontists must understand SDB, its consequences, associated risks, comorbidities, and the correlation of these conditions with craniofacial dysmorphisms and dysfunctions [13, 44–46]. In the present study, however, 51.6% of orthodontics professors reported that the training of orthodontists in understanding OSA was deficient, while 41.6% considered it adequate. These findings are relevant, particularly considering that the evaluated professors are themselves orthodontists and educators of future dentists, whose investigative skills could play a crucial role in the early screening of OSA patients.

Although the present study was primarily diagnostic and did not evaluate or implement pedagogical strategies, we acknowledge that the absence of innovative teaching proposals may be viewed as a limitation, particularly in the context of journals with an educational focus. Nevertheless, our findings provide a foundation for the design of future interventions, such as integrated curricula, problem-based learning, clinical simulation, and interdisciplinary programs in dental sleep medicine. In this regard, this study represents an initial step in identifying curricular gaps and highlighting opportunities for pedagogical innovation.

### Study limitations

This study has some methodological limitations that should be acknowledged. Data were collected between March and October 2022, and curricular structures may have evolved since then. The use of self-reported questionnaires may have introduced recall and social desirability biases, affecting the accuracy of reported teaching practices. The response rate of 51.7% among contacted institutions also limits the representativeness of the sample, and programs or faculty with greater interest in SDB may have been more likely to participate, potentially overestimating curricular inclusion or awareness. Additionally, no formal web-survey reporting guideline was prospectively adopted, which may limit the reporting of some platform-specific metrics; however, key methodological elements (sampling flow, instruments, and descriptive analyses) are reported in detail.

Although restricting the analysis to dental schools participating in the 2019 National Student Performance Exam provided a relevant national overview of programs with graduating students, the sample may not fully reflect all Brazilian dental curricula. The focus on orthodontics professors, whose specialty is directly linked to craniofacial growth and risk factors for SDB, offered valuable insight into a key group involved in airway-related care, but limited the perspectives from other disciplines. In addition, orthodontics professors were recruited through course coordinators, characterizing a two-stage recruitment process. This approach may have introduced selection bias, since coordinators who agreed to participate may have been more engaged with the topic and more likely to provide contacts of faculty members with greater interest in SDB. Additionally, the absence of complementary qualitative methods (e.g., interviews or curriculum review) restricted opportunities for data triangulation, although coordinators’ responses still contributed institution-level information.

Despite these limitations, this survey represents the first nationwide effort to map the teaching of SDB in Brazilian dental schools. The findings highlight important curricular gaps and align with international evidence of insufficient training, underscoring the need for broader, methodologically diverse research to guide future improvements in dental sleep medicine education.

### Implications for practice and future research

The study highlights critical curricular gaps and points to clear directions for improvement. Expanding curricular workload, fostering interdisciplinary integration, and providing faculty development opportunities are essential steps to strengthen training in this area. For clinical practice, these measures would enhance dentists’ preparedness to identify and contribute to the management of SDB, with broader implications for public health. Future research should move beyond situational diagnosis and advance toward the design, implementation, and evaluation of innovative pedagogical strategies - such as problem-based learning, clinical simulations, and integrated modules - that can effectively incorporate SDB into undergraduate dental training.

## Conclusion

This nationwide study revealed that SDB remains minimally addressed in Brazilian dental curricula, with limited and predominantly theoretical workloads and few opportunities for clinical practice. Although coordinators and faculty acknowledge its relevance, curricular integration is fragmented and insufficient. Strengthening the inclusion of SDB/OSA content may benefit from expanding instructional hours, investing in targeted faculty development, and promoting interdisciplinary approaches with sleep medicine specialists.

Building on these findings, the results highlight opportunities for the structured integration of SDB/OSA topics into undergraduate dental education, supported by interprofessional initiatives and faculty training programs. Future research should advance beyond situational diagnosis to develop, implement, and evaluate innovative pedagogical strategies - such as problem-based learning, clinical simulations, and integrated modules - that may effectively support the incorporation of SDB teaching into dental curricula.

## Supplementary Information

Supplementary Material 1.

Supplementary Material 2.

Supplementary Material 3.

Supplementary Material 4.

## Data Availability

The datasets generated and analyzed during the current study are publicly available in the Open Science Framework (OSF) repository, under the title “Aggregated Dataset – Teaching of Sleep-Disordered Breathing in Brazilian dental schools: a nationwide study” (Leal CPB, Flório FM, 2025). DOI: [10.17605/OSF.IO/JTMZ7](10.17605/OSF.IO/JTMZ7). The repository contains aggregated, non-identifiable data corresponding to all variables presented in Tables 1, 2, 3, 4 and 5 of the manuscript, as well as the full versions of the questionnaires used in the study, in both Portuguese (original format) and English (translated version).

## Abbreviations

SDB: Sleep-disordered breathing
OSA: Obstructive sleep apnea
ENADE: National Student Performance Exam
MEC: Ministry of Education of Brazil
CBCT: Cone-beam computed tomography
