# Supplementary material for: Teaching of sleep-disordered breathing in Brazilian dental schools: a nationwide study
Source: BMC Med Educ. 2026 Jun 3;26:1269. doi: 10.1186/s12909-026-09497-w (PMC13449327; doi:10.1186/s12909-026-09497-w)
Supplement: Supplementary file 1 [file 12909_2026_9497_MOESM1_ESM.docx]

Coordinator Questionnaire (English Translation)
Original language: Portuguese (Brazil)

The study of sleep disorders has gained importance within modern Dentistry, as these conditions coexist with several problems routinely evaluated by dentists, such as bruxism, mouth breathing, temporomandibular disorders, maxillary atresia, and other craniofacial anomalies. This correlation increases the responsibility of general practitioners in the early diagnosis, screening, and referral of affected patients.

In this context, there is a growing need for greater attention to Dental Sleep Medicine in undergraduate dental curricula and for better postgraduate training in this area, which would substantially benefit patients, particularly those with obstructive sleep apnea syndrome (OSA).

The objective of this study was to analyze the profile of sleep-disordered breathing (SDB) teaching in Brazilian undergraduate dental programs, investigating whether these topics are included in the curriculum, how they are presented, the theoretical-practical workload, and whether specific sleep-related questions or questionnaires are used during clinical anamnesis.

We kindly ask you to respond sincerely to this brief questionnaire or forward it to the appropriate department. Thank you very much for your consideration and prompt response. The compiled results will be shared with participants after data analysis.

**Section 1 – Institutional profile**
1. Region of the institution:
 ( ) North
 ( ) Northeast
 ( ) Central-West
 ( ) Southeast
 ( ) South

2. Type of institution:
 ( ) Public (Federal, State, or Municipal)
 ( ) Private

3. Course duration (in years): ___ years
4. Total course workload (in hours): ___ hours

**Section 2 – Curriculum structure**
5. Does your curriculum include any topics related to sleep-disordered breathing (SDB)?
 ( ) Yes	( ) No

6. Is there a specific course, module, or discipline on Dental Sleep Medicine (DSM)?
 ( ) Yes	( ) No

7. If yes, indicate the total number of teaching hours allocated to this content: ___ hours

8. How is the topic addressed within the curriculum?
 ( ) Theoretical classes
 ( ) Practical classes / clinical activities
 ( ) Seminars / case discussions
 ( ) Elective course
 ( ) Extension or continuing education activities

9. Which topics related to SDB are covered in your curriculum? (More than one option may be selected)
 [ ] Sleep bruxism
 [ ] Snoring
 [ ] Obstructive sleep apnea (OSA)
 [ ] Sleep hygiene
 [ ] Orofacial growth and airway
 [ ] Diagnosis and management of SDB
 [ ] Others: _______________________

10. Are there any professors trained in Dental Sleep Medicine in your institution?
 ( ) Yes	( ) No

11. In your opinion, what are the main challenges for including this topic in the undergraduate curriculum?
 [ ] Lack of trained faculty
 [ ] Lack of time in the curriculum
 [ ] Lack of awareness or priority among coordinators
 [ ] Limited physical space or infrastructure
 [ ] Lack of institutional or administrative support
 [ ] Limited access to patients or diagnostic resources
 [ ] Other: _______________________

**Section 3 – Institutional initiatives and support**
12. Does your institution offer any extension or continuing education programs related to SDB?
 ( ) Yes	( ) No

13. If yes, specify which: _______________________

14. Does your institution have a Dental Sleep Medicine (DSM) or SDB clinic?
 ( ) Yes	( ) No

15. Is your institution involved in any partnerships or collaborations in this area (e.g., with medical schools, hospitals, or sleep centers)?
 ( ) Yes	( ) No	( ) Not applicable

16. If yes, describe briefly: _______________________

17. In your opinion, should the topic of SDB be formally incorporated into undergraduate dental curricula?
 ( ) Yes	( ) No	( ) Not sure

**Notes for Reproduction**
This questionnaire was originally developed in Portuguese by the research team and administered electronically via an online survey platform. Minor adaptations were made to ensure semantic equivalence in English.
