# Supplementary material for: Teaching of sleep-disordered breathing in Brazilian dental schools: a nationwide study
Source: BMC Med Educ. 2026 Jun 3;26:1269. doi: 10.1186/s12909-026-09497-w (PMC13449327; doi:10.1186/s12909-026-09497-w)
Supplement: Supplementary file 2 [file 12909_2026_9497_MOESM2_ESM.docx]

**Questionário Coordenadores**

O ensino dos Distúrbios do sono tem ganhado espaço dentro da Odontologia atual, principalmente porque coexistem com vários problemas já avaliados pelos dentistas, como o bruxismo, a respiração bucal, a Disfunção temporo- mandibular (DTM), a atrésia da maxila e outras anomalias craniofaciais entre outros. Esta correlação aumenta a responsabilidade do profissional generalista no diagnóstico precoce, triagem e encaminhamento do paciente para um possível tratamento. Neste contexto, existe a necessidade de mais atenção para a Odontologia do Sono nos currículos odontológicos, bem como uma melhor formação de pós-graduação neste domínio, o que beneficiaria muito os pacientes, sobretudo aqueles com Síndrome da apneia obstrutiva do sono (SAOS). Este estudo tem por objetivo analisar o perfil do ensino dos distúrbios respiratórios do sono (DRS) nos cursos de graduação em Odontologia brasileiros, observando primeiramente se estão presentes no currículo, como são apresentados e a carga horária teórico-prática, se há perguntas específicas sobre os DRS nos prontuários das clínicas e/ou se são aplicados aos pacientes questionários específicos sobre os distúrbios do sono durante a anamnese. Solicitamos que você responda sinceramente a este breve questionário de perguntas ou o envie aos respectivos departamentos para resposta. Muito obrigado antecipadamente por sua consideração e resposta rápida. Retornaremos os resultados para você quando tabulados.

1. Em que região geográfica esta sua instituição?

o Nordeste

o Sudeste

o Centro Oeste

o Norte

o Sul

2. Sua instituição é privada ou pública?

o Privada

o. Pública

3. Qual é a carga horária do curso de Odontologia?

o Menos de 4000 horas

o 4000 horas

o Entre 4001 e 5000 horas

d. Mais de 5000 horas

4. Quantos anos têm o curso de Odontologia?

o 1 a 5 anos

o 6 a 10 anos

o 11 a 15 anos

o 16 a 20 anos

o Mais de 20 anos

5. O Sr(a)possui uma Clínica de Odontologia do Sono em sua Instituição?

o Sim

o Não

6. Algum departamento/ disciplina de sua Instituição engloba o estudo dos distúrbios do sono/ odontologia do sono

o Sim

o Não

7. Se a resposta for positiva, por favor marque qual departamento.

o Endodontia

o Ortodontia

o Prótese

o Cirurgia

o Odontopediatria

o Periodontia

o Outro:___________

8. Sua Instituição possui corpo docente treinado em distúrbios do sono/Odontologia do sono

o Sim

o Não

9. Que desafios o Sr(a) enfrentou para implementar o estudo dos distúrbios do sono/Odontologia do sono em sua instituição?

o Aprovação / suporte administrativo/

o Espaço físico

o Não há um número suficiente de professores treinados em Odontologia do sono

o Sem desafios

o Outro: ___________

10. Quantas horas de aula teórica ou clínica são destinadas ao ensino de tópicos distúrbios do sono/ Odontologia do sono em cada ano do curso?

| Primeiro ano | 0 | 1 | 2 | 3 | 4 | 5 | 6 | 7 | 8 | >8 |
| --- | --- | --- | --- | --- | --- | --- | --- | --- | --- | --- |
| Segundo ano | 0 | 1 | 2 | 3 | 4 | 5 | 6 | 7 | 8 | >8 |
| Terceiro ano | 0 | 1 | 2 | 3 | 4 | 5 | 6 | 7 | 8 | >8 |
| Quarto ano | 0 | 1 | 2 | 3 | 4 | 5 | 6 | 7 | 8 | >8 |
| Quinto ano  ( se houver) | 0 | 1 | 2 | 3 | 4 | 5 | 6 | 7 | 8 | >8 |

11. Como é desenvolvido este ensino:

| Primeiro Ano | Didática | Laboratório Pré-clínico | Clínica |
| --- | --- | --- | --- |
| Segundo Ano |  |  |  |
| Terceiro Ano |  |  |  |
| Quarto Ano |  |  |  |
| Quinto Ano  ( se houver) |  |  |  |

12. Quais os tópicos relacionados a Odontologia do sono são abordados? (Por favor, marque todas as opções aplicáveis).

o Ronco primário

o Síndrome de resistência das vias aéreas superiores

o Apneia obstrutiva do sono

o Transtorno de comportamento REM

o Síndrome da perna inquieta

o Insônia

o Distúrbio de movimento periódico das pernas

o Mudanças de fase do sono

o Bruxismo do sono

13.Quais terapias para distúrbios respiratórios do sono são discutidas? (Por favor, marque todas as opções aplicáveis).

o CPAP

o. Terapia com Aparelho Oral

o Terapias cirúrgicas

o Cirurgia bucomaxilofacial (por exemplo, avanço mandibular)

o Abordagens ortodônticas e ortopédicas

o Outro:________________

14.Que outros tópicos são discutidos? (Por favor, circule todas as opções aplicáveis).

o Uso de instrumentos de questionário de sono e sonolência diurna

o Consequências médicas de distúrbios respiratórios do sono não tratados

o Consequências psicossociais de distúrbios respiratórios do sono não tratados

o Polissonografia

o Atendimento coordenado com médicos do sono e otorrinolaringologistas

15. A sua instituição tem programas de educação continuada em distúrbios do sono/Odontologia do sono?

o Sim

o Não

16. Se respondeu sim à pergunta anterior, com que frequência são oferecidos cursos de educação continuada sobre distúrbios do sono /Odontologia do sono?

o A cada 1 a 3 meses

o A cada 4 a 6 meses

o A cada 7 a 9 meses

o A cada 10 a 12 meses

17. Se respondeu sim a pergunta anterior, o(s) seu(s) programa(s) de curso(s) de educação continuada sobre distúrbios do sono /Odontologia do sono tem um mínimo de 25 horas?

o Sim

o Não
