# Supplementary material for: Teaching of sleep-disordered breathing in Brazilian dental schools: a nationwide study
Source: BMC Med Educ. 2026 Jun 3;26:1269. doi: 10.1186/s12909-026-09497-w (PMC13449327; doi:10.1186/s12909-026-09497-w)
Supplement: Supplementary file 3 [file 12909_2026_9497_MOESM3_ESM.docx]

**Professor Questionnaire (English Translation)
Original language: Portuguese (Brazil)**

Dear Professor,

Your participation in this survey is very important to help us outline the current panorama of sleep-disorders education - especially obstructive sleep apnea (OSA) - in undergraduate dental programs. These data will contribute to reflections on the role of orthodontics professors in integrating this topic into dental education and on how this aligns with national curricular guidelines.

**Section 1 – Professional profile**
1. Gender:
 ( ) Female
 ( ) Male
 ( ) Prefer not to answer

2. Region of your institution:
 ( ) North
 ( ) Northeast
 ( ) Central-West
 ( ) Southeast
 ( ) South

3. Type of institution where you teach:
 ( ) Public (Federal, State, or Municipal)
 ( ) Private

4. How long ago did you graduate in Dentistry?
 ___ years

5. Do you hold a postgraduate degree?
 ( ) Yes
 ( ) No

6. If yes, specify:
 ( ) Lato sensu specialization
 ( ) Master’s degree
 ( ) Doctoral degree

7. Is your postgraduate training in Orthodontics?
 ( ) Yes
 ( ) No

8. How long have you been teaching Orthodontics?
 ___ years

9. Do you work exclusively as a university professor?
 ( ) Yes
 ( ) No

10. Do you also work in private practice or other professional activities?
 ( ) Yes
 ( ) No

**Section 2 – Knowledge and exposure to Dental Sleep Medicine (DSM)**
11. Have you ever heard of sleep-disordered breathing (SDB)?
 ( ) Yes
 ( ) No

12. Have you ever heard of Dental Sleep Medicine (DSM)?
 ( ) Yes
 ( ) No

13. Have you received any information or training on DSM or SDB?
 ( ) Yes
 ( ) No

14. If yes, what was the main source?
 ( ) Undergraduate course
 ( ) Postgraduate course
 ( ) Scientific event or congress
 ( ) Continuing education course
 ( ) Scientific literature (articles, books)
 ( ) Social media or informal sources
 ( ) Other: _______________________

15. Do you know of any dental schools that include topics on DSM or SDB in their curricula?
 ( ) Yes
 ( ) No
 ( ) Not sure

16. Do you feel prepared to teach topics related to DSM or SDB?
 ( ) Yes, very prepared
 ( ) Partially prepared
 ( ) Not prepared

17. Have you ever referred a patient suspected of having obstructive sleep apnea (OSA) to a sleep physician or another professional?
 ( ) Yes
 ( ) No

**Section 3 – Perceptions regarding teaching and clinical management of SDB**

18. In your opinion, how important is it for general dentists to receive training in SDB and OSA?
 ( ) Very important
 ( ) Important
 ( ) Of little importance
 ( ) Not important

19. In your opinion, how important is it for orthodontists to receive training in SDB and OSA?
 ( ) Very important
 ( ) Important
 ( ) Of little importance
 ( ) Not important

20. How do you rate the current level of training that dental students receive on SDB and OSA?
 ( ) Excellent
 ( ) Very good
 ( ) Good
 ( ) Regular
 ( ) Poor
 ( ) None

21. Which topics do you usually address during Orthodontics classes? (More than one option may be selected)
 [ ] Craniofacial growth and development
 [ ] Airway assessment
 [ ] Mouth breathing
 [ ] Snoring
 [ ] Sleep bruxism
 [ ] Obstructive sleep apnea (OSA)
 [ ] Other: _______________________

22. Do you believe that dentists play a role in the screening and referral of patients with OSA?
 ( ) Yes
 ( ) No
 ( ) Not sure

23. Do you ask your patients (children, adolescents, or adults) about snoring or sleep-related complaints during anamnesis?
 ( ) Always
 ( ) Sometimes
 ( ) Rarely
 ( ) Never

24. Do you collaborate with sleep physicians or other professionals in managing patients with SDB or OSA?
 ( ) Yes
 ( ) No

25. Have you ever participated in a continuing education activity on DSM or SDB?
 ( ) Yes
 ( ) No

26. If not, would you be interested in participating?
 ( ) Yes
 ( ) No
 ( ) Maybe

**Section 4 – Attitudes and perceptions**
27. Please indicate your level of agreement with the following statements:

 a) SDB should be addressed in undergraduate dental curricula.
 [ ] Strongly agree	[ ] Agree	[ ] No opinion	[ ] Disagree	[ ] Strongly disagree

 b) Orthodontists play a key role in the early identification of OSA.
 [ ] Strongly agree	[ ] Agree	[ ] No opinion	[ ] Disagree	[ ] Strongly disagree

 c) Dentists can contribute to the multidisciplinary management of patients with SDB.
 [ ] Strongly agree	[ ] Agree	[ ] No opinion	[ ] Disagree	[ ] Strongly disagree

 d) There is sufficient information about SDB in current dental curricula.
 [ ] Strongly agree	[ ] Agree	[ ] No opinion	[ ] Disagree	[ ] Strongly disagree

 e) The inclusion of SDB topics would improve students’ clinical training.
 [ ] Strongly agree	[ ] Agree	[ ] No opinion	[ ] Disagree	[ ] Strongly disagree

**Notes for Reproduction**
This questionnaire was originally developed in Portuguese by the research team and administered electronically via an online survey platform. Minor adaptations were made to ensure semantic equivalence in English.
