# Supplementary material for: Teaching of sleep-disordered breathing in Brazilian dental schools: a nationwide study
Source: BMC Med Educ. 2026 Jun 3;26:1269. doi: 10.1186/s12909-026-09497-w (PMC13449327; doi:10.1186/s12909-026-09497-w)
Supplement: Supplementary file 4 [file 12909_2026_9497_MOESM4_ESM.docx]

**Questionário docentes**

Senhor (a) professor (a),

Sua resposta a este questionário é muito importante para traçarmos o panorama do ensino sobre os distúrbios do sono, em especial, a AOS, na graduação em odontologia. Com estes dados, esperamos obter informações, que nos ajudem a refletir sobre o papel atual do professor de ortodontia na inserção do tema ensino de graduação, bem como, a fundamentação de sua realidade educacional segundo as diretrizes curriculares nacionais.

**INFORMAÇÕES GERAIS**

1) Sexo

a. Homem

b. Mulher

2) Idade____anos

3) Formação

a. Especialização

b. Mestrado

c. Doutorado

d. Pós-doutorado

4) Ano de Graduação na Faculdade de Odontologia__________

5) É especialista em ortodontia?

a) Sim

b) Não

6) Se sim, quantos anos de Especialista em Ortodontia____ anos

7) Sua universidade possui uma cadeira específica para a disciplina de ortodontia?

a) Sim

b) Não

8) Se não, em qual disciplina é dado o conteúdo de ortodontia? _________

9) Quantos anos como professor da disciplina de ortodontia, ou de outra disciplina que englobe a carga horária de ortodontia? ______ anos

10) Atua como professor em cursos de pós-graduação em ortodontia?

a) Sim

b) Não

11) Possui algum tipo de capacitação em Odontologia do sono?

a) Especialização

b) Aperfeiçoamento

c) Cursos de extensão com mínimo de 30 horas

d) Palestras

e) Outros

12) Há alguma abordagem aos Distúrbios do sono em sua disciplina do curso de graduação?

a) Sim

b) Não

13) Se sim, quantas horas de ensino? _______ horas

14) Que desafios o Sr(a) enfrentou para implementar o estudo dos distúrbios do sono/Odontologia do sono em sua disciplina?

a. Aprovação / suporte administrativo

b. Espaço físico

c. Treinamento específico em Odontologia do sono

d. Sem desafios

e. Outra, por favor, especifique

15) Como classifica a importância do ensino dos distúrbios do sono na graduação:

a) Muito importante

b) Importante

c) Não é importante

d) Não sei

16) Como você avalia a formação do dentista no entendimento da AOS:

a) Ótima

b) Muito boa

c) Regular

d) Deficiente

17) Como classifica a importância do ensino da distúrbios do sono na especialização em ortodontia:

a) Muito importante

b) Importante

c) Não é importante

d) Não sei

18) Como você avalia a formação do ortodontista no entendimento da AOS:

a) Ótima

b) Muito boa

c) Regular

d) Deficiente

**CONHECIMENTO DA AOS**

Triagem e diagnóstico

1) Qual é o padrão ouro para o diagnóstico da AOS?

a) questionário STOP – BANG

b) Polissonografia

c) História do caso

d) Não sei

2) Você está familiarizado com algum questionário específico do sono para a triagem prévia de pacientes com risco de AOS?

a) Sim

b) Não

3) Se sim, qual? ___________________________________________________

4) Você utiliza algum questionário específico do sono para a triagem prévia de pacientes com risco de AOS?

a) Sim

b) Não

5) Se sim, qual? ___________________________________________________

6) Durante a sua anamnese, você faz alguma pergunta relacionada a AOS?

a) Sim

b) Não

7) Durante a sua anamnese, você mede o IMC dos seus pacientes?

a) Sim

b) Não

c) Às vezes

d) Nunca

8) Durante a sua anamnese, você mede a circunferência do pescoço dos seus pacientes?

a) Sim

b) Não

c) Às vezes

d) Nunca

9) O diagnóstico definitivo da AOS pode ser feito por um dentista?

a) Sim

b) Não

c) Não sei

**TRATAMENTO E ENCAMINHAMENTO da AOS**

1) Com relação ao tratamento da AOS qual das alternativas abaixo não está correta?

a) A AOS leve não requer tratamento

b) A AOS leve é tratada com aparelhos intraorais

c) A AOS moderada e severa é tratada com CPAP e cirurgia Ortognática

d) A AOS severa é tratada com cirurgia Ortognática

e) Não sei

2) Quem prescreve os aparelhos intraorais para os pacientes?

a) O dentista

b) O médico do sono

c) Não sei

3) Você já encaminhou algum adulto para o médico do sono por suspeita de AOS?

a) Sim

b) Não

c) Não sei

4) Você classificaria a expansão rápida da maxila como uma terapia para a AOS em crianças?

a) Sim

b) Não

c) Não sei

5) Você já encaminhou alguma criança para o otorrinolaringologista por suspeita de AOS?

a) Sim

b) Não

c) Não sei

6) Você já recebeu alguma criança do otorrinolaringologista para tratamento de AOS?

a) Sim

b) Não

c) Não sei

**INFORMAÇÕES GERAIS**

1) A AOS é mais comum entre:

a) Homens

b) Mulheres

c) Ambos

d) Não sei

2) Roncar é um sintoma observado em pacientes com AOS?

a) Sim

b) Não

c) Não sei

3) A prevalência da AOS aumenta com a idade?

a) Sim

b) Não

c) Não sei

4) Você classificaria os aparelhos ortopédicos de avanço dos maxilares como uma terapia para a AOS em crianças?

a) Sim

b) Não

c) Não sei

**FATORES DE RISCO**

1) Os fatores que contribuem com AOS são:

a) Obesidade

b) Hipertensão

c) Obesidade e/ou Hipertensão

d) Não sei

2) Você acha que o desenvolvimento anormal da maxila e mandíbula pode ser um fator de risco para AOS?

a) Sim

b) Não

c) Não sei

3) Você acha que existe algum tipo facial mais susceptível para desenvolver AOS?

a) Dolicofacial

b) Mesofacial

c) Braquifacial

d) Não há

e) Não sei

4) Adenóides aumentadas podem ser um fator de risco para AOS?

a) Sim

b) Não

c)Não sei

5) Na sua anamnese, você utiliza algum sistema para avaliar as amigdalas e tonsilas (Mallampati, etc.)?

a) Sim

b) Não

c) Não sei

**TRIAGEM E DIAGNÓSTICO**

1) Os dentistas desempenham um papel importante no diagnóstico e tratamento da AOS

a) Concordo

b) Concordo fortemente

c) Não tenho opinião formada

d) Discordo

e) Discordo fortemente

2) Quando o dentista identifica o hábito de bruxismo em seu paciente, é sua função indagar sobre ronco e AOS.

a) Concordo

b) Concordo fortemente

c) Não tenho opinião formada

d) Discordo

e) Discordo fortemente

3) É importante que o dentista pergunte sobre o padrão de sono de seu paciente durante a anamnese?

a) Concordo

b) Concordo fortemente

c) Não tenho opinião formada

d) Discordo

e) Discordo fortemente

**ABORDAGEM INTERDISCIPLINAR**

1) Você acha que durante a graduação o currículo odontológico deve incluir informações sobre AOS e o papel do dentista neste problema de saúde?

a) Concordo

b) Concordo fortemente

c) Não tenho opinião formada

d)Discordo

e) Discordo fortemente

2) A triagem para detecção de AOS do paciente deve ser uma parte obrigatória do exame clínico para os dentistas?

a) Concordo

b) Concordo fortemente

c) Não tenho opinião formada

d) Discordo

e) Discordo fortement

3) Dentista e médicos do sono devem lidar juntos com pacientes com AOS?

a) Concordo

b) Concordo fortemente

c) Não tenho opinião formada

d) Discordo

e) Discordo fortemente

4) Se o dentista encontrar estruturas anatômicas orais anormais, ele deve fazer uma investigação mais aprofundada para AOS e encaminhar o paciente para um médico do sono.

a) Concordo

b) Concordo fortemente

c) Não tenho opinião formada

d) Discordo

e) Discordo fortemente

**TRIAGEM, DIAGNÓSTICO E TRATAMENTO**

1)Você já perguntou ao seu paciente sobre a história do sono após observar os desgastes em seus dentes?

a) Sim

b) Não

2) Você já investigou AOS em pacientes que apresentaram histórico de ronco?

a) Sim

b) Não

3) Se sim, como? __________________________________________________

4) Você encaminhou seu paciente ao médico para diagnóstico de distúrbios do sono depois de perceber achados orais relacionados à AOS?

a) Sim

b) Não

5) Você já fabricou algum aparelho oral para tratar seu paciente com AOS?

a) Sim

b) Não

6) Você já participou de algum curso sobre manejo de pacientes com SAOS?

a) Sim

b) Não

7) Você teria interesse em frequentar um curso de manejo odontológico de doenças bucais relacionadas ao sono?

a) Sim

b) Não
